# Dr. H. A. Ramsay, of Ga., and the Am. Med. Association

**Published:** 1851-10

**Authors:** 


### EDITORIAL DEPARTMENT.

*Dr. H. A. Ramsay, of Ga., and the Am. Med. Association.* — During the summer of 1850, Dr. Ramsay, a practitioner residing at Raysville, Ga., communicated for the Philadelphia Medical Examiner, an article purporting to be a Report of a statistical analysis of 473 recorded cases of obstetrics, which he represented to have occurred in his practice during a period of about ten years; stating, also, that he had attended about two hundred additional cases, of which he had taken no notes.

Some account of these statistics, it appears, was embodied in the Report on obstetrics as originally made to the American Medical Association, at the meeting in Charleston, in May last.

After the Report just mentioned was read to the Association, a member of the Association, Dr. Robertson, of S. C., moved that the Report be recommitted to the Committee, stating, as the reason for the motion, that "it was made to give the chairman an opportunity of striking out the statistics of Dr. Ramsay, as it had been asserted, on the most undoubted authority, that they were not reliable." This language is quoted from a statement subsequently written and published by Dr. Robertson. The motion of Dr. R., prevailed, and the statistics of Dr. Ramsay were stricken out by the chairman of the committee on obstetrics. The foregoing facts appeared substantially in the minutes of the meeting of the Association as published in most of the Medical Journals of the country.

That a member of the profession, who, it seems, was not present at the meeting, should be accused in so public a manner of forging statistical data, and, as the result showed, not only accused, but condemned unheard, and without any investigation, and his name stigmatised in the official record of the Association, struck us, at the time, as extraordinary; and we then noted the subject in our list of editorial topics, but afterward concluded to await the development of farther facts.

Shortly after the meeting of the Association, we observed in several medical journals a card from Dr. Ramsay, soliciting a suspension of judgment by the profession, until he had time to make his defence. We did not insert this card in our columns, having received no request to that effect, and not having quoted in our Journal that part of the Report of the proceedings of the Association which reflected upon the character of Dr. R.

Subsequently we received a printed document addressed to the members of the American Medical Association, by Dr. Robertson, author of the public charge of fraud upon Dr. Ramsay; and recently a pamphlet by Dr. R. has been forwarded to us by the author.

Both parties having thus come before the public, we deem it proper, and our duty, as a journalist, to notice the matter. In doing so, we wish to state distinctly that we have no knowledge of, nor have we had any communication with either of the parties, directly, or through the intervention of friends, and the only facts bearing on the subject which are in our possession, are those that appear in the two pamphlets just referred to.

Two questions are involved in the subject, to wit, 1st. Was the charge brought against Dr. Ramsay, by Dr. Robertson, proper under the circumstances? 2d. Is there sufficient evidence to prove the truth or falsity of the charge?

With reference to the first of these questions, we give the statement of the circumstances which led Dr. Robertson to make the charge, in his own words:

"Soon after the delegates to the late meeting of the American Medical Association, in our city, commenced to assemble, reference was made by some one — I do not now recollect who — in a casual conversation, to the Obstetrical Statistics of Dr. H. A. Ramsay, of Georgia. I had not, up to that time, seen the tables referred to.

"Dr. Reyburn, the chairman of the committee on Medical Literature, stated that he had referred to them in his report, and would like to have further and authentic information on the subject. I introduced him to Dr. J. J. Robertson and Dr. Andrews, of Washington, Georgia, who were in attendance as visitors to the meeting of the Association, though not delegates, and, subsequently, to other medical gentlemen from Georgia, who were delegates. The result of his conference with these gentlemen, was a determination, on the part of Dr. Reyburn, to erase all allusion to the contributions of Dr. Ramsay from his report, before presenting it to the Association; which, I understand, was done. This occurred some days previous to the presentation of either the report of Dr. Reyburn, or that of Dr. Storer.

"From this time, the reliability of the statistics of Dr. Ramsay became the subject of free conversation among many members of the Association. It was ascertained, from the gentlemen mentioned above, and some of the delegates from Georgia, that he had been a practitioner only about ten years, and not continuous in one place. When this was considered, in connection with the number of cases reported, amounting, with the 200 of which no particular notes were taken, to 672, it struck many old and experienced practitioners as an unusually large number of cases for the first ten years' practice, in a portion of country not densely populated, and in which there were the usual number of competitors for professional patronage. For ten years' practice, admitting that he was, immediately upon graduationg, introduced into a general obstetrical practice — which is not usually the case — would give an average of 67 cases, and a fraction over, per year; or an average of more than one case a week for the entire ten years.* If the cases comprised those which came under his notice in six years, this would give 112 cases, and a fraction over per year; or an average of two and a fraction over, for each week in the six years. This simple analysis, made by several persons in casual conversation upon the subject, struck every one — and particularly practitioners from the country — with much force. One gentlemen, from Philadelphia, remarked that he had considered the statistics unreliable upon reading them, at the time of their publication, without knowing any thing of the contributor, or the circumstances he had heard here. It was stated by many practitioners from the country, and by the two gentlemen first named, who were personally acquainted with Dr. Ramsay, and who practised in the adjoining county — and frequently in Dr. Ramsay's neighborhood — that it was not generally customary to employ a physician in obstetrical cases among the blacks on plantations, unless in cases of difficulty, or apprehended difficulty; and that it was by no means a universal custom to employ them among whites, many still adhering to the old custom of sending for the midwife. It was not a difficult matter for each one to refer to his own experience in the practice of obstetrics for a series of years, or for a single year; and when they did, and then considered the number of cases reported by Dr. Ramsay — the length of time he had been in practice — the nature of the country and population in which he had practiced, and the necessary interruption occasioned by a removal from one location to another — the conviction seemed to be forced upon every mind that the statistics carried with them the strongest internal evidence of their unreliability, aside from any other considerations. But, further, I was informed, by a member of the Georgia State Medical Society, a delegate to the American Medical Association, whose name can be given if necessary, that at the annual meeting of that body, previous to the meeting of the National Association in Charleston, Dr. Cooper read a report, by appointment, on Obstetrics, and referred to Dr. Ramsay's tables as authority for certain views. The next day he begged leave to retract what reference he had made to Dr. Ramsay's cases, as he had

---

* When this analysis was made, it was on the statement of several persons, that there were about 200 cases which had not been recorded, not included in the 473. Since writing to Dr. DeSaussure, I have again referred, more particularly, to Dr. Ramsay's article, and find the following remarks : — " In the early part of my professional career, I carefully recorded all my difficult obstetrical cases, leaving the simple labors to fare for themselves ; hence, the reader will find an aggregate of 444 cases only, when, in reality, I am confident I have attended 600 or more." This, leaving out the " *more*," will vary the results of the analysis a mere trifle. I have, however, deemed it but fair to quote from " the record."

heard they were *not reliable.* The permission to alter the report was granted.*

"I have made the above statement, to show you what had taken place in relation to Dr. Ramsay's statistics, before the subject was brought before the Association, and that, too, in the Medical Society of his own state.

"When Dr. Storer's report on Obstetrics was read, on the afternoon of the 7th of May, it was found that the statistics in question had been incorporated in it. The following is an extract from the official report of the proceedings of the Association, contained in Vol. 6th, No. 4, of the Charleston Medical Journal and Review, at page 576, and which is the only accurate and authentic report that I have seen.

"'Dr. Storer, of Ms., chairman of the committee on Obstetrics, presented and read the report of the committee.

"'On motion of Dr. Robertson, of South Carolina, the report was recommitted to the committee, and made the special order for the morning session.'

"At the time of making this motion, I stated, distinctly, that it was made to give the chairman an opportunity of striking out the statistics of Dr. Ramsay, as it had been asserted, on the most undoubted authority, that they were not reliable. I mentioned that the parties, upon whose authority the motion had been made, were in the hall, and the chairman of the committee would have an opportunity of conferring with them, and other gentlemen from Georgia, before he acted in the premises. Although there was a large delegation from Georgia in attendance at the time, yet not one objected to the motion, or uttered a word in defence of the statistics of the author, although many of them were personally acquainted with Dr. Ramsay. Moreover, I have been since informed, by a member from Georgia, that the Georgia delegation held a conference in relation to the motion, previous to the final adoption of the report, and decided not to interfere in the matter.

"Among the delegates from Georgia, who were personally acquainted with Dr. Ramsay, were Doctors Ford, Garvin, and P. F. Eve, who were professors in the Medical College at which Dr. Ramsay graduated.

"The above, sir, constitutes the authority upon which my motion to recommit was made. Under the circumstances, I considered it a duty which I owed to the chairman of the committee and the Association, to offer the resolution, particularly as Dr. J. J. Robertson and Dr. Andrews were not delegates, and, therefore, could not do so; although they both hold themselves responsible for the authority upon which it was made, and will, cheerfully, respond to any call which Dr. Ramsay may think proper to make on them.

"In the part which I have taken in the matter, I have been actuated by no personal feelings towards Dr. Ramsay, as I have no acquaintance whatever with him. With similar facts before me, in relation to the statistics of any other medical writer, I should have felt bound, as a member of the Association, to have pursued the same course. In making the motion, on the authentic information which had been given, I have done no more towards

* It is important to remark, that Dr. Andrews and Dr. J. J. Robertson, were not privy to the charge of "*unreliability,*" made in the Georgia State Medical Society, as they were *not at that meeting,* nor are they members of that body. These two sources of uthority are, therefore, separate and distinct, having no connection with each other.

inflicting an injury upon Dr. Ramsay than those who assented, on the same authority, to the erasure of his statistics — no more than Dr. Cooper and the Medical Society of his own State, who requested and was granted permission by the Society to erase them from his report; and I should be happy to be informed of the code of ethics that can deprive any body of medical men of the right to entertain, and act upon, such a motion, under similar circumstances."

After reading carefully Dr. Robertson's statement, we are constrained to say that, in our judgment, he was not justified in the remarks made by him to the Association. We arrive at this opinion without any reference to the actual truth or falsity of the accusation of Dr. Ramsay. The question now is, not whether the charges have been subsequently disproved or sustained, but whether they were warranted by facts at that time in the possession of Dr. Robertson. These facts are sufficient only for a *presumption* that the statistics were not reliable. They do by no means *prove* that the data were forged. Private and professional character we hold to be too sacred to be impeached, on such an occasion, and in such a manner, except on the strongest and clearest evidence. The rumors, surmises, and calculations obtained from a few of the professional neighbors of the accused, do not constitute evidence of that description. In saying this we do not mean to imply that Dr. Robertson acted from improper motives. We see no reason to doubt that he acted conscientiously, and from a sense of duty. But to effect the object he had in view, which was to protect the interests of science from what he presumed to be *false facts*, it was not necessary to prefer a public accusation against an absent member of the profession. The reasons for discrediting the statistics might have been made privately to the committee, and their erasure from the report accomplished quite as well without running the risk of doing an innocent person great injustice and injury. It is much to be regretted that the remarks by Dr. Robertson were recorded by the secretaries of the Association; and it is surprising that at the reading of the minutes the portion embracing these remarks were not expunged. We shall be sorry to see them in the printed Transactions. The question as to the propriety of the course pursued by Dr. Robertson covers broader ground than that occupied by this particular instance. On this account we dwell the more upon it. Every member of the profession who has a reputation or a character to loose, or who is laboring to acquire an honorable distinction, is interested therein. Let the propriety of this precedent be recognised, and who is safe? The purest and most valued in the profession may have jealous or malignant neighbors who would spare no pains to circulate slanders and insinuations, if they might hope to bring their aspersions to bear upon the objects of their spite, as heavily as, justly or unjustly, the stigma of falsehood and fraud has fallen upon Dr. Ramsay.

With respect to the *second* question, relating to the truth or falsity of the charge brought against Dr. Ramsay, we shall not presume to offer a judicative decision. It is however due to Dr. Ramsay, not only to suspend an unfavorable judgment until he is proved guilty; but, considering the publicity of the charge against him, the confident assurance with which it was made, and the channel through which it comes to the medical public, viz., the proceedings of the Am. Med. Association, it is but an act of justice to examine, candidly and carefully, the testimony submitted in the defence. In the printed appeal by Dr. Ramsay, addressed to the medical profession (which we must say is rather unfortunate in the style of composition) he gives the certificates of several medical practitioners, and others residing in his neighborhood, attesting that the circumstances stated by Dr. Robertson to militate against the reliability of the statistics, were not as represented in the quotation which we have given at length — in other words, that the author of the statistics has not been moving from place to place; that the part of the country is thickly populated; that he has had but little competition in midwifery practice, and that on this account, and from its not being the custom in his neighborhood, as in some others, to employ midwives, his practice in this department has been quite extensive. These gentlemen, or some of them, bear testimony to the honesty and honorable character of Dr. Ramsay, and express the belief that the number of cases which Dr. Ramsay professes to have attended, is not remarkable. This kind of evidence depends, for its force, upon the character of the persons whose names are attached to the certificates. Most of them are probably unknown to members of the profession residing at a distance. The name of but one is familiar to us, and this one from his contributions to medical journals.

Dr. Ramsay offers to exhibit the note book, containing the records of the cases, to any persons, or to publish an attested copy of it at Philadelphia. We will take the liberty of suggesting that were he to submit the original records to a few persons well known to the profession, who should be satisfied with respect to the internal evidence of good faith thereby afforded, the exculpation would probably be deemed more satisfactory by those who are equally unacquainted with the standing of Dr. Ramsay, and those of his professional neighbors who have come to his support.

As we have already said, we have no sympathies derived from personal knowledge of either of the authors of the two pamphlets before us, but admitting, as we are bound in common charity to do, the possibility, if not probability, that Dr. Ramsay is laboring under a false imputation of professional dishonesty, it does not require an extraordinary degree of sensibility not to be altogether indifferent to the painful position in which he has been placed. If unjustly assailed, we sincerely hope he may succeed in establishing the fact beyond a question; but if, on the other hand, the accusation be just, it is due to truth and science that all doubt on the subject be removed.

